# Efficacy of different doses of corticosteroids in treating severe COVID-19 pneumonia

**DOI:** 10.1186/s12985-024-02345-7

**Published:** 2024-03-26

**Authors:** Ge zhang, Lin Su, Wenwen Wu, Qing Qiao, Shuncui Gao, Yan Zhang, Yanmei Zhang

**Affiliations:** Department of Respiratory and Critical Care Medicine, The Third Affiliated Hospital of Shandong First Medical University, Jinan Fourth People’s Hospital, Shandong Jinan, 250000 China

**Keywords:** Severe COVID-19 pneumonia, Corticosteroid, Inflammatory factors, Mortality, Chest CT features

## Abstract

**Background:**

To investigate the efficacy of different doses of corticosteroids in treating severe coronavirus disease 2019 (COVID-19) pneumonia.

**Methods:**

Between May 01, 2023, and June 20, 2023, 48 patients with severe COVID-19 pneumonia were treated at the Department of Respiratory and Critical Care Medicine of Jinan Fourth People's Hospital. The observation group (21 patients) received standard care and high-dose corticosteroids, (high-dose group). The control group (27 patients) received standard care and low-dose corticosteroids (low-dose group). We collected baseline data and recorded inflammatory marker levels after 3 days of treatment, body temperature recovery time, length of stay, and 28-day all-cause mortality. The results of outpatient follow-up were recorded after 1 month.

**Results:**

There were no significant differences in 28-day mortality and length of stay. The number of days it took for body temperature to return to normal in the high-dose group was less than in the low-dose group. The high-dose group had significantly more reduced inflammatory factors (C-reactive protein (CRP), interleukin-6 (IL-6). A total of 20 discharged patients were given 8–16 mg of methylprednisolone, depending on chest computed tomography (CT) and clinical symptoms after 1 month; in all discharged patients using oral corticosteroids, CT features improved.

**Conclusion:**

High-dose corticosteroids had a significantly positive effect on the reduction of inflammatory factors and shortening body temperature recovery time. In the treatment of severe COVID-19 pneumonia, early administration of high-dose, short-course corticosteroids should be implemented.

## Highlights

The highlights of the paper are:

1. High-dose corticosteroid can shorten the recovery time of body temperature.

2. High-dose corticosteroid can better reduce inflammatory factors (CRP IL-6).

3. Oral hormones in discharged patients with severe COVID-19 pneumonia are beneficial.

4. Initial dosage and course of corticosteroid might be varied case by case.

## Background

Coronavirus disease 2019 (COVID-19) is a disease caused by severe acute respiratory syndrome coronavirus 2 (SARS-CoV-2); it was first reported in Wuhan, China, before spreading as a global pandemic. On January 12, 2020, the novel coronavirus was named "2019 Novel Coronavirus" by the World Health Organization (WHO) [1]. COVID-19 has caused more than 5 million deaths [2]. Because mutation of the virus has continued, it remains a major global health challenge. China has a large number of elderly people who have concomitant diseases such as diabetes and high blood pressure, meaning that the proportion of people with severe COVID-19 pneumonia is higher, and its treatment needs significant attention. Although the pathophysiology of severe COVID-19 pneumonia is still not completely known, it has been well established that deregulation of the host immune response and a massive inflammatory response, known as a “cytokine storm”, play an important role in causing pathological damage to the lungs [3, 4]. Plasma sampling from patients with severe COVID-19 has revealed high circulating levels of immune-inflammatory markers such as interleukin (IL)-6, IL-1β, IL-2, IL-7, IL-17, and tumor necrosis factor (TNF)-α; this is also the reason behind the anti-inflammatory treatments used for COVID-19 pneumonia. Jinan, in Shandong Province, had its first wave of infections after the COVID-19 control policy was lifted on December 5, 2022. With antibodies declining for 4–6 months, a second peak of infection occurred again in mid-April, 2023. Systemic corticosteroids are considered effective in treating severe or critical COVID-19 patients in both Chinese and international consensus and guidelines [5–7]. According to the "Diagnosis and Treatment Plan for Novel Coronavirus Infection (Trial Version 10)" on January 5, 2023 [8] and the "Diagnosis and Treatment Plan for Severe Cases of Novel Coronavirus Infection (Trial Version 4)" on January 13, 2023, issued by the General Office of the National Health Commission, there has been a uniform and clear recommendation for using corticosteroids in the treatment of COVID-19; dexamethasone (6 mg daily for up to 10 days) is usually recommended. However, based on the outcomes of clinical trials treating COVID-19 patients since 2020, the starting time, initial dosage, and course of corticosteroid might vary case by case; Professor Huang Hui of Peking Union Medical College Hospital also talked about this in an article published in May 2023 [9]. Systematic retrospective analysis of Cochrane databases for 2021 and 2022 found that hormones can improve all-cause mortality in hospitalized COVID-19 patients; a high-dose (dexamethasone ≥ 12 mg once daily or other equivalent hormones) group was compared to a low-dose (dexamethasone 6–8 mg once daily or other equivalent hormones) group, and the former reduced the all-cause case fatality rate within 30 days [10, 11]. Pinzón et al. [12] found that COVID-19 patients treated with high-dose and long-course hormones (methylprednisolone at 250–500 mg daily for 3 days, followed by prednisolone at 50 mg daily for 7–10 days) had more benefits; time for disease improvement was shortened, the chance of transfer to the intensive care unit (ICU) was reduced, and C-reactive protein (CRP) levels were significantly reduced. Granholm et al. [13] found that, compared with dexamethasone 6 mg daily, increasing the dose to 12 mg daily increased benefits and lowered hormone-related adverse reactions in COVID-19 patients with severe hypoxia (nasal catheter oxygen inhalation ≥ 10 L/min). Based on the above, patients with severe COVID-19 pneumonia in our department were given different doses of corticosteroids, and we observed the clinical efficacy in the two groups to provide more clinical evidence for the use of high doses of corticosteroids.

## Methods

### Study population

Patients were enrolled in the study if they met all of the inclusion criteria: patients aged at least 18 years admitted to hospital, diagnosis of COVID-19 confirmed by positive nasopharyngeal polymerase chain reaction testing (RT-PCR) for SARS-CoV-2 infection; oxygenation index (PaO2/FiO2) not more than 300, and bilateral pulmonary infiltrates on chest imaging (> 50%) indicative of COVID-19 severe pneumonia. Exclusion criteria were: the use of other immunomodulatory therapies, such as IL-6 or JAK inhibitors, and patients treated with tocilizumab or baricitinib (as these two immunosuppressants were available at our pharmacy).

### Intervention

Patients allocated to the high-dose corticosteroid group received dexamethasone at 10–15 mg (or other equivalent hormones) daily for 3–5 days, followed by dexamethasone at 5–7.5 mg (or other equivalent hormones) for 3–5 days, followed by dexamethasone at 3 mg until discharge. The control group was given low-dose corticosteroids, usually dexamethasone at 6 mg (or other equivalent hormones) once daily for 5–10 days, followed by dexamethasone at 3 mg (or other equivalent hormones) until discharge if sooner. All participants received standard treatment for COVID-19, including prone position ventilation, antiviral therapy, anticoagulation, maintenance of water and electrolyte balance, nutritional support, symptomatic treatment of underlying diseases, anti-infective therapy if necessary, respiratory support therapy (nasal tube oxygen, high-flow nasal cannula, non-invasive ventilator assisted ventilation, or invasive mechanical ventilation), and stress ulcer prophylaxis.

### Data collection

Data were carefully recorded from medical files in a standardized data collection form by an attending. After review and confirmation by our chief physician, the collected data were transferred into an electronic database. The information collected included: demographic data, underlying diseases, clinical signs and symptoms at enrollment, laboratory results at enrollment, in-hospital COVID-19 therapies received, changes in inflammatory indicators (CRP, IL-6, ferritin) after 3 days of treatment (day 4), number of days corticosteroid therapy was received, and clinical outcomes. For discharged patients, outpatient follow-up (mainly for chest computed tomography (CT) review) was completed after 1 month.

Ground glass opacity(GGO) and fibrosis were classified to evaluate the high-resolution computed tomography (HRCT) findings [14]. HRCT assessment categorized the condition as improving, stable, and aggravating. Improvement and aggravation were defined by a decrease or increase of at least 10% of the overall disease extent, respectively, while stability was defined by changes of less than 10% in either direcction [15]. Two experienced radiologists blinded to the patients’ conditions independently assessed the HRCT images.

### Statistical analyses

All data were analyzed using SPSS software version 26. The qualitative variables were described using frequency (%) and compared by Chi-square or Fisher’s exact tests. The variables with or without normal distribution were reported as the mean with standard deviation (SD), or median (percentile 25–75), respectively. The unpaired sample t-test was performed to compare quantitative variables between observation and control groups. *p*-values < 0.05 were considered significant.

#### Ethical approval

This study was approved by the Ethics Committee of Jinan Fourth People’s Hospital (approval number: LL20230035). Informed consent was waived by the ethical committee since the study was observational and there was no diagnostic or therapeutic intervention outside the usual clinical practice or the need for additional collection of biological samples.

## Results

**Fig. 1:**
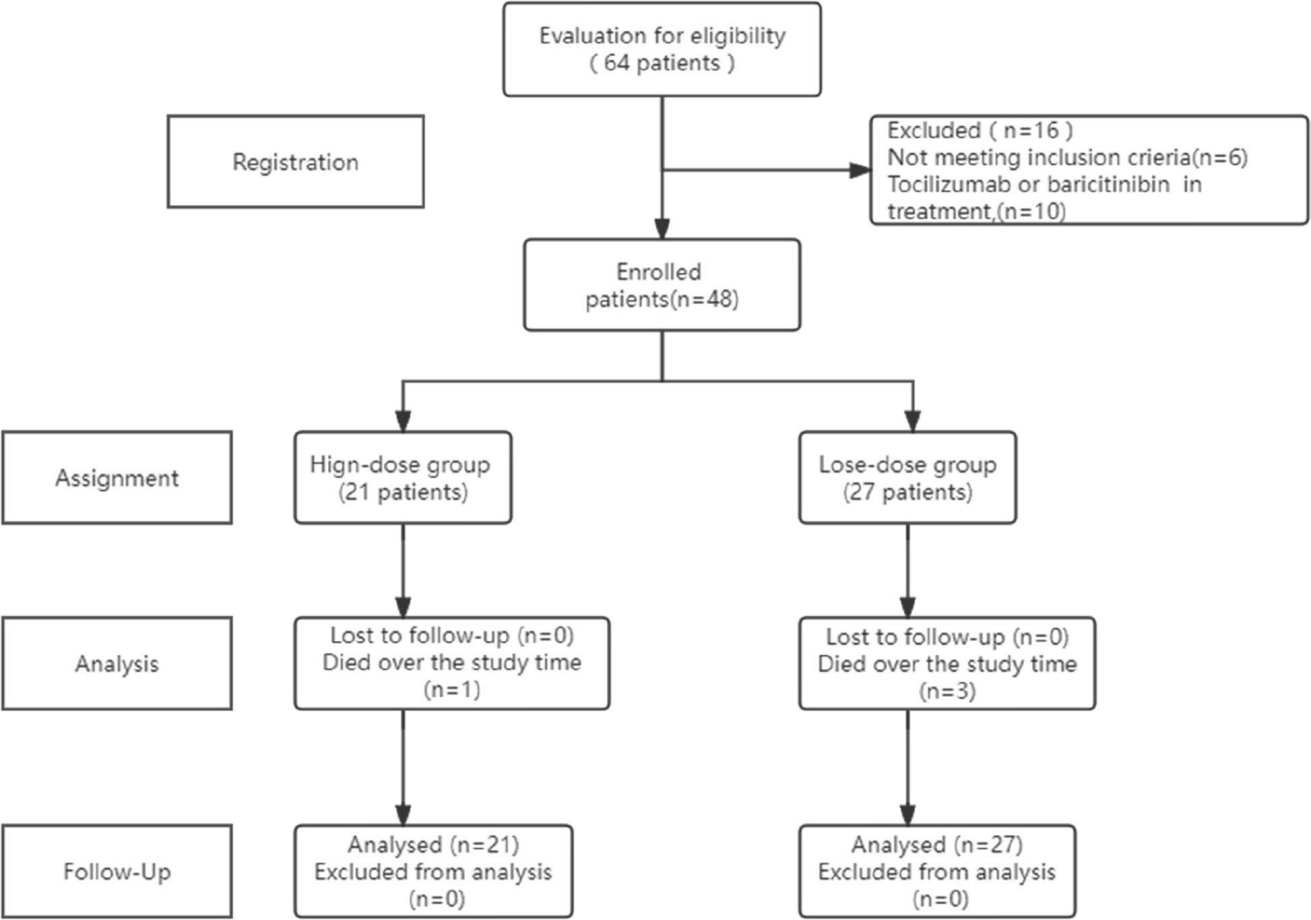
Flow chart of the study

**Fig. 2:**
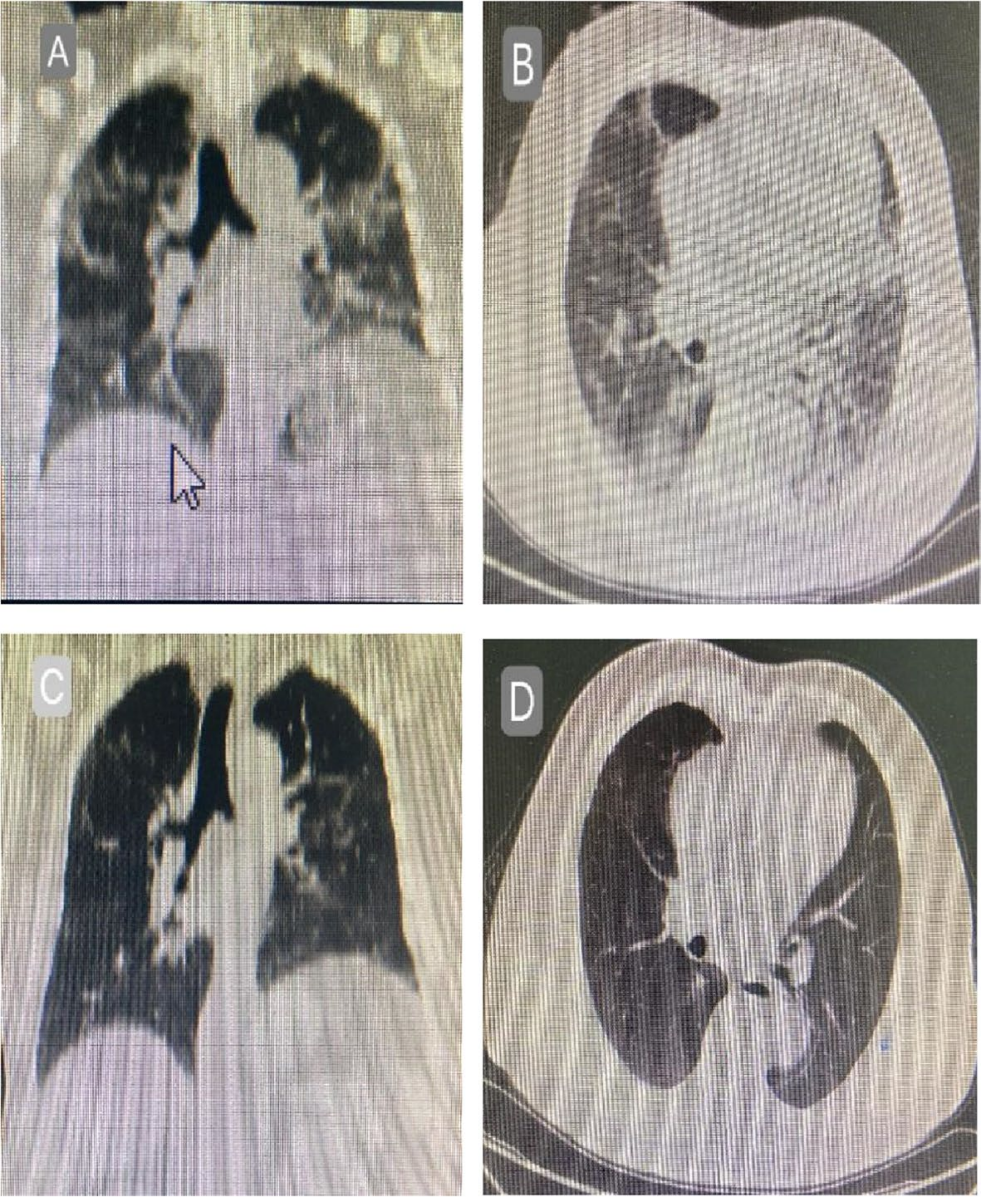
Coronal reconstruction and axial image from CT imaging of the chest acquired when discharged in a 73-year-old man (**A** and **B**) shows a radiological pattern with predominant peribronchial and perilobular dense consolidation mild traction bronchiectasis of the airways. Follow-up CT imaging of the chest acquired after 1 month of oral prednisolone (**C** and **D**) shows residual ground glass and a little subpleural consolidation. As shown in the image below)

The flow chart of the study protocol is shown in Figs. 1 and 2. Between May 01, 2023, and June 20, 2023, 64 patients with a diagnosis of severe COVID-19 pneumonia were assessed for study eligibility; six were excluded based on the inclusion and exclusion criteria at the discretion of the treating physicians**,** as needed by the condition. Ten patients used tocilizumab or baricitinib and were also excluded. In total, 48 patients satisfied the inclusion criteria; based on the initial corticosteroid dosage, they were divided into the high-dose (21 patients) and low-dose (27 patients) groups. Fig.2 is a CT contrast of a typical follow-up case(at discharge and one month after discharge).

### Demographics and baseline characteristics

The baseline demographic and clinical data of the enrolled patients are summarized in Table 1. There were no statistical differences in the baseline data among the two groups. The mean age of the patients was 74.25 ± 9.27 years, those over 65 years old made up 81% of the population, and 68.75% (33 out of 48 patients) were male. The estimated mean ± SD of lymphocytes was 0.75 ± 0.37 × 10^9^ cells/L, which was below normal (0.8–3.5 × 10^9^ cells/L). The estimated mean ± SD of blood sugar was 8.57 ± 4.17 mmol/L. The majority of patients (79.1%) had at least one comorbidity; those over 65 years old typically had 2–3 comorbidities. The most common comorbidities were cardiovascular diseases (47.9%) hypertension (45.8%), and diabetes (37.5%), and there were no significant differences between the groups. According to HRCT findings, all patients had bilateral interstitial pneumonia at baseline; almost all had initial ground-glass opacity in both lungs, followed by ground-glass opacity with consolidation (similar to organizing pneumonia).

**Table 1:** Baseline demographic and clinical features of the observation and control groups

Variable	High-dose group (21 patients)	Low-dose group (27 patients)	*P*-value
Age, years, mean ± SD	74.90 ± 2.079	73.93 ± 1.775	0.721
Sex (M/F), n (%)	14/7(66.7/33.3)	19/8(70.4/29.6)	0.784
Temperature, °C, mean ± S D	38.05 ± 0.95	37.89 ± 0.79	0.361
PaO2/FiO2 ratio, mmHg, mean ± SD	201.19 ± 14.21	209.25 ± 11.08	0.65
White blood cell, 109/L, mean ± SD	6.629 ± 0.807	6.613 ± 0.816	0.990
Neutrophils, 109/L, mean ± SD	4.99 ± 0.78	5.43 ± 0.78	0.697
Lymphocyte, 109/L, mean ± SD	0.67 ± 0.06	0.79 ± 0.08	0.17
Platelet, 109/L, mean ± SD	178.57 ± 14.95	186.74 ± 14.93	0.69
CRP, mg/L, mean ± SD	94.98 ± 13.69	88.76 ± 7.74	0.43
IL-6,pg/ml, mean ± SD	60.69 ± 14.08	32.09 ± 7.37	0.06
Ferritin,μg/L, mean ± SD	301.30 ± 60.75	220.43 ± 42.4	0.25
Creatine kinase, U/L, mean ± SD	1.56 ± 0.15	1.75 ± 0.21	0.49
Creatinine, U/L, mean ± SD	64.11 ± 3.61	73.89 ± 5.56	0.17
Alanine aminotransferase, U/L,mean ± SD	25.37 ± 3.2	33.51 ± 5.2	0.22
Glutamate aminotransferase, mean ± SD	27.13 ± 1.83	27.37 ± 2.37	0.93
Blood sugar, mmol/L, mean ± SD	8.61 ± 0.87	8.53 ± 0.34	0.95
Cardiovascular disease, n (%)	10(47.6%)	13(48.1%)	0.82
Diabetes, n (%)	8(38.1%)	10(37.0%)	0.85
Respiratory disease, n (%)	4(19.0%)	5(18.5%)	0.78
Neurologic disorder, n (%)	6(28.6%)	8(37.0%)	0.70
hypertension, n (%)	8(38.1%)	14(51.8%)	0.20

*CRP* C-reactive protein, *IL-6* Interleukin-6

The estimated mean ± SD of the PaO2:FiO2 ratio at enrolment was 201.19 ± 14.21 and 209.25 ± 11.08 in the high-dose and low-dose groups, respectively. The baseline levels of the inflammatory factors (CRP, IL-6, ferritin) were elevated, and there was no significant difference between groups (*p* = 0.43, 0.06, and 0.25, respectively).

### Primary and secondary clinical efficacy outcomes

Tables 2 show the comparison of the primary and secondary clinical efficacy outcomes among the study groups. Although patients in the high-dose group had lower overall mortality during the 28-day follow-up period than those in the low-dose group (4.76 vs. 11.11%), no statistically significant differences were found (Table 2; *p* = 0.43). The number of days until body temperature returned to normal was 2.00 ± 0.46 in the high-dose group, significantly shorter than the 4.48 ± 1.04 in the low-dose group (Table 2; *p* = 0.04). The mean ± SD of ventilator-free days was 12.19 ± 1.30 in the high-dose group and 13.92 ± 1.40 in the low-dose group, with no statistically significant differences (*p* = 0.38). During treatment, eight fungal infections occurred in the high-dose group (38.1%), but compared with the low-dose group, the chance of fungal infection was not significantly increased (Table 2; *p* = 0.53).

**Table 2:** Primary and secondary study clinical outcomes up to day 28

Variable	Ventilator-free days, mean ± SD	28-day mortality, n (%)	Days of temperature return to normal mean ± SD	Number of fungal infection n (%)
High-dose group	12.19 ± 1.30	1(4.76%)	2.00 ± 0.46	8(38.1%)
Low-dose group	13.92 ± 1.40	3(11.11%)	4.48 ± 1.04	8(29.6%)
t/ × 2	0.885	0.623	1.974	0.381
*P*-value	0.38	0.43	0.04	0.53

As shown in Table 3, the high-dose group had better-controlled inflammation than the low-dose group; levels of CRP and IL-6 were statistically different (*p* < 0.05). Of the patients discharged, 20 were given corticosteroids (8 to 16 mg of methylprednisolone); doses varied depending on CT findings (such as more than 15% ground-glass opacity, subpleural and peribronchial linear dense consolidation, varying degrees of traction bronchiectasis) and clinical symptoms. A month later, 40 patients completed outpatient follow-up, and four were lost to follow-up (including one patient taking oral hormones). In all 19 cases using oral corticosteroids, CT features improved; chest CT had a fainter ground-glass pattern in the same distribution, and the density of consolidation decreased uniformly after treatment. In these 19 patients, we did not see progression to any fibrosis.

**Table 3:** The inflammation factors (after treatment of the first three days)

Variable	High-dose group (21 patients)	Low-dose group (27 patients)	t	*P*-value
CRP mean ± SD	27.44 ± 7.64	46.05 ± 4.37	1.707	0.01
IL-6mean ± SD	19.46 ± 6.31	30.17 ± 9.41	1.143	0.02
Ferritin mean ± SD	407.11 ± 80.69	368.04 ± 84.36	-0.598	0.43

## Discussion

The effectiveness of corticosteroid therapy in reducing mortality in COVID-19, especially in severely ill patients, has been demonstrated, but the optimal time and dose to provide the maximum benefits are undetermined. Our study observed the effects of different doses of corticosteroids on patients with severe COVID-19 pneumonia. The RECOVERY trial showed a clear reduction in 28-day mortality with the use of 6 mg dexamethasone once daily for up to 10 days in patients with COVID-19 requiring oxygen therapy [16]. In our study, high-dose vs low-dose corticosteroids did not significantly change 28-day mortality for severe COVID-19 pneumonia. The number of enrolled patients was small, however, which could potentially lead to bias in outcomes. More patients must be enrolled in similar treatment comparisons to determine the appropriate hormone dose. Additionally, during the initial screening process, 10 patients were excluded that had been treated with baricitinib or tocilizumab, because we were unable to assess any possible interactions between immunomodulatory treatments and corticosteroids. Consequently, these patients were excluded, the effect observed may be an overestimate of the true effects of corticosteroids.

In the early stages of COVID-19, white blood cells are normal or decreased and lymphocyte count is reduced. In our study, the baseline data of patients showed that the mean lymphocyte count (0.67–0.79 × 10^9^/L) was below the normal mean (0.8–4.0 × 10^9^/L). The data show that the early determination of lymphocytes and T lymphocyte subsets helps judge the severity and prognosis of severe COVID-19 pneumonia and can be used to guide clinical treatment [17]. As patients recover, lymphocytes rise to normal. Examining the influence of COVID-19 on T lymphocyte subsets is also one of our team's future research projects.

This clinical study found that the average blood glucose level of patients was about 8.5 mmol/L. COVID-19 can cause blood glucose to rise, the pathological mechanism of which is unknown. Insulin resistance is the prevalent cause of hyperglycemia, independent of corticosteroid treatment; in addition, SARS-CoV-2 destroys fat cells and triggers adipose tissue dysfunction. Because of this, patients with COVID-19 have lower levels of adiponectin [18]. Previous studies also found that hyperglycemia is a strong poor prognostic factor for COVID-19. Elevated baseline blood glucose levels, as well as the use of corticosteroids after admission, inevitably lead to extended hospital stays [19], indicating that this phenomenon requires attention from clinicians.

In this clinical study, the fungal infection rate was high, with the infection rate in the high-dose group reaching 38.1%; this indicates that clinicians must pay attention to screening for fungal infections and make timely treatment plans. Because of long hospital stays coupled with the use of corticosteroids and IL-6 antagonists, some patients developed superinfection. COVID-19-associated pulmonary aspergillosis (CAPA) and COVID-19-associated mucormycosis (CAM) have been increasingly reported during the COVID-19 pandemic. Co-pathogenesis of COVID-19 and fungal infections are thought to occur because of the high inflammatory status and immune dysregulation during severe COVID-19 pneumonia [20, 21], as well as the disruption of the tissue barrier of the respiratory epithelium following COVID-19. Because of the cytokine storm that occurs in COVID-19, the diagnosis of fungal infections is challenging as markers are often indistinguishable from this. Most patients with severe COVID-19 are elderly, and the combination of viral and fungal infection undoubtedly aggravates the difficulty of treatment; because of this, prompt detection and treatment of fungal infections in patients with COVID-19 pneumonia is crucial to improve patient outcomes. Complications induced by the treatment with corticosteroids are a major concern, including steroid diabetes, secondary infections, peptic ulcers, gastrointestinal bleeding, increased blood pressure, and sodium and water retention; secondary infections are of particular concern. However, no severe complications caused by corticosteroids were observed in this study, including nosocomial infections.

The natural history of recovery from COVID-19 remains unknown. The most common radiological pattern of COVID-19 is bilateral ground-glass opacification with or without consolidation at the bilateral inferior pleural border. Organized pneumonia patterns are described in many cases [22]. Following COVID-19, some patients are left with both radiological inflammatory lung disease and persistent decreased function.

Overall, treatment with oral corticosteroids was well tolerated in this patient group and was associated with rapid and significant improvement [23]. At discharge, corticosteroid therapy was recommended in 20 patients because of the presence of poor oxygen absorption and the absence of symptom improvement. A rapid wean over 1 month was chosen because the driver of inflammation was absent when patients were discharged from the hospital. In some cases, the reduced speed was slowed further by the outpatient doctor. After one month, oral corticosteroids led to an improvement in inflammation on CT scans.

## Conclusion

The present data suggest that high-dose corticosteroids improve the reduction of inflammatory factors and shorten body temperature recovery time in patients with severe COVID-19 pneumonia. Oral hormones after discharge were beneficial for the improvement of pulmonary inflammation, indicating they could be used to avoid progression to pulmonary interstitial fibrosis. However, this was not a randomized control trial, and the results were biased because of the low number of patients enrolled. We look forward to more multicenter, randomized, double-blind trials to support these results.

## Data Availability

The datasets used or analyzed during the current study are available from the first author on reasonable request.

## Abbreviations

COVID-19: Coronavirus disease 2019
SARS-CoV-2: Severe acute respiratory syndrome coronavirus 2
WHO: World Health Organization
IL: Interleukin
TNF: Tumor necrosis factor
ICU: Intensive care unit
CRP: C-reactive protein
RT-PCR: Polymerase chain reaction testing
CT: Computed tomography
GGO: Ground glass opacity
HRCT: High-resolution computed tomography
SD: Standard deviation
